# Embargo on American Cattle

**Published:** 1894-11

**Authors:** 


### EMBARGO ON AMERICAN CATTLE.

The recent action of Germany and Denmark in placing an embargo upon the importation of American live cattle and dressed meats, on the grounds of the danger of importation of Texas fever, is uncalled for, and will not bear the scrutiny that such action should afford. We are very sure that these countries have not taken these steps on the recommendation of their high veterinary authorities, who are all too intelligent and too well informed to be ignorant of the fact that the danger in this direction is the least of any of the diseases, which are infectious and contagious, when but a small portion of Texas cattle are shipped directly abroad, most of them being shipped to higher altitudes of our own country, and there fattened, during which time they gain a freedom and immunity from the danger of this disease, and thus carry with them no danger to any live stock interests abroad. We have not noticed a single quotation in any foreign journal of any outbreak of Texas fever that might be considered the result of any imported live stock. It seem rather a retaliatory measure, and we would have admired their honesty and sincerity much more, if they would have said truthfully that this was one of the results that might be anticipated in adopting tariff measures that militated against these countries. It requires a log stretch of one's imagination to conceive of an educated and intelligent country taking such action, when they must necessarily know that they would be open to the most severe and just criticism for such a movement.

